# Low Dietary n6/n3 Ratio Attenuates Changes in the NRF 2 Gene Expression, Lipid Peroxidation, and Inflammatory Markers Induced by Fructose Overconsumption in the Rat Abdominal Adipose Tissue

**DOI:** 10.3390/antiox10122005

**Published:** 2021-12-16

**Authors:** Petra Roškarić, Marcela Šperanda, Tomislav Mašek, Donatella Verbanac, Kristina Starčević

**Affiliations:** 1 Department of Chemistry and Biochemistry, Faculty of Veterinary Medicine, University of Zagreb, Heinzelova 55, 10000 Zagreb, Croatia; petra.roskaric@vef.hr; 2 Department of Animal Science, Faculty of Agriculture, University of Osijek, Vladimira Preloga 1, 31000 Osijek, Croatia; marcela.speranda@fazos.hr; 3 Department of Animal Nutrition and Dietetics, Faculty of Veterinary Medicine, University of Zagreb, Heinzelova 55, 10000 Zagreb, Croatia; tomislav.masek@vef.hr; 4 Department of Medical Biochemistry and Hematology, Faculty of Pharmacy and Biochemistry, University of Zagreb, A. Kovačića 1, 10000 Zagreb, Croatia; donatella.verbanac@pharma.unizg.hr

**Keywords:** adipose tissue, metabolic syndrome, oxidative stress, DHA, fatty acids

## Abstract

The objective of this study was to examine the benefits of different n6/n3 polyunsaturated fatty acid ratios on the lipid metabolism, insulin resistance, and oxidative stress in the adipose tissue of rats fed a high-fructose diet. Male and female rats were divided into four groups: a control group (CON) (n6/n3 ratio ~7), a high-fructose group (HF) (n6/n3 ratio ~7), an N6-HF group (n6/n3 ratio ~50), and the DHA-HF group (n6/n3 ratio ~1, with the addition of docosahexaenoic (DHA) and eicosapentaenoic (EPA) acid). The CON group received plain water and the HF group received 15% fructose in their drinking water. Fructose induced an increase in the content of serum triglycerides, serum cholesterol, and HOMA-IR index. Among the fatty acids, elevated proportions of C18:1n9 and C16:1n7, as well as an increase in total monounsaturated fatty acid (MUFA), were found in the adipose tissue of the HF group. Fructose treatment also changed oxidative parameters, including a marked increase in the serum malondialdehyde (MDA) content. Meanwhile, DHA supplementation caused a significant decrease in the serum MDA concentration in comparison with the HF group. In addition, DHA/EPA supplementation attenuated oxidative stress by increasing NRF 2 gene expression. Fructose treatment also significantly decreased the adiponectin level, while DHA supplementation ameliorated it. The changes observed in this trial, including the decrease in the content of DHA and EPA, the decreased EPA/ARA ratio, and the increase in the expression of inflammatory genes, are characteristics of the low-grade inflammation caused by fructose treatment. These changes in the rat adipose tissue could be prevented by dietary intervention consisting of DHA supplementation and a low n6/n3 ratio.

## 1. Introduction

Increased fructose intake caused by overconsumption of soft drinks plays an important role in the development of metabolic syndrome, dyslipidaemia, and adipose tissue dysfunction [1,2]. Abdominal obesity, dyslipidaemia, hyperglycaemia, hypertension, and low-grade inflammation are the hallmarks of metabolic syndrome [3].

A key component of the development of metabolic syndrome is dysfunction of the lipid metabolism characterised by changes in lipogenesis and fatty acid concentrations in different tissues. Adipose tissue represents the main storage of fatty acids (FA) in the form of triglycerides and is also an active endocrine organ with different signalling and regulatory properties that affect local and systemic metabolic responses [4]. Hypertrophy of adipocytes caused by obesity leads to the altered secretion of adiponectin and inflammatory cytokines [5].

Polyunsaturated fatty acids (PUFAs) are the major component of the membrane phospholipids which influence the membranes’ physical properties and cell functions [6,7]. It is generally considered that a change in the whole body’s fatty acid composition can be attributed to different sources of fatty acids, mainly diet, de novo lipogenesis (DNL), and bioconversion. Fatty acids derived from the diet or generated de novo are bioconverted by a series of highly regulated steps of desaturation, elongation, and β-oxidation, into different saturated and unsaturated fatty acids [8,9]. The bioconversion rate of linolenic acid into important n3 PUFA, eicosapentaenoic acid (EPA), and docosahexaenoic acid (DHA) is very low, and therefore, a percentage of these fatty acids must be obtained by diet [10]. A large number of recent studies suggest that n3 PUFA, EPA, and DHA, have a wide range of beneficial biological effects, including prevention of obesity, insulin resistance, decreasing oxidative stress, and anti-inflammatory effects [11]. The alteration of n6/n3 polyunsaturated fatty acids ratio in favour of n3 PUFA has an important role in the prevention and treatment of metabolic disorders, such as metabolic syndrome [12].

Adipose tissue is a reservoir of polyunsaturated fatty acids for other tissues, and the alteration of its fatty acid composition may have important effects on the other tissues, and animal and human health in general. Therefore, we aimed to characterise the efficiency of diets with different amounts of EPA and DHA, and different n6/n3 ratios on the prevention of the pathological changes in fructose-induced metabolic syndrome. In addition, we investigated whether this nutritional manipulation is sex specific.

## 2. Materials and Methods

### 2.1. Animals, Study Design, and Experimental Diets—Experimental Design

**Figure 1:**
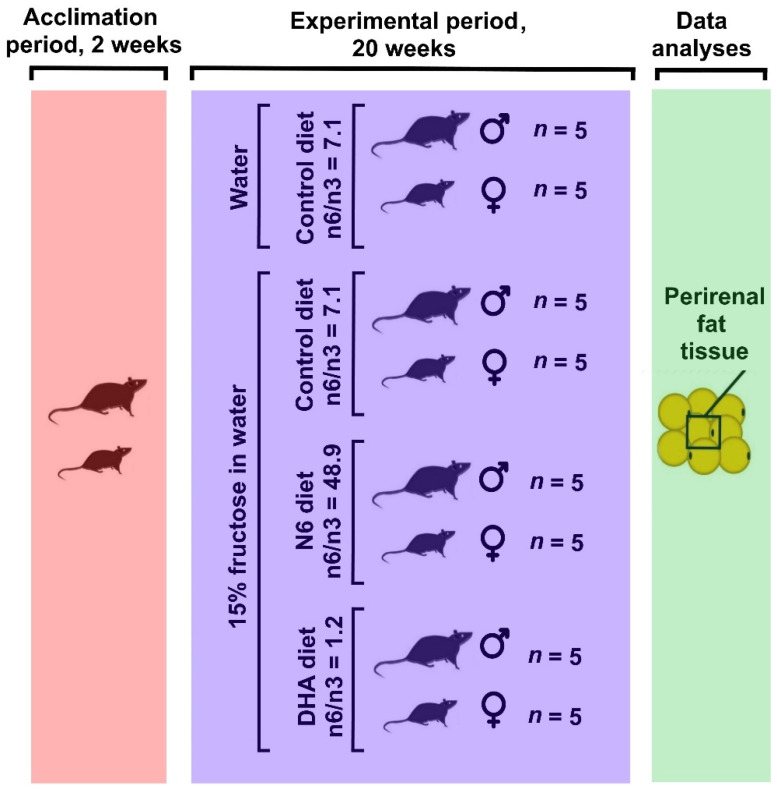
Graphical representation of the experimental groups of rats. The rats were divided into four groups based on their diet.

All experiments were approved by the Croatian National Ethics Committee and the Ministry of Agriculture, Republic of Croatia (Authorisation EP 107/2017), and conducted in accordance with the Croatian Animal Welfare Act. The experiment was performed on 40 Wistar HAN rats (20 males 177 ± 6 g and 20 females 140 ± 4 g). At the beginning of the experiment, after two weeks’ acclimation, the rats were assigned to the different dietary treatment and fructose supplementation into control (CON) or high-fructose (HF) groups. The control group was given plain water, while the HF group received 15% fructose in the drinking water. The HF group was additionally divided on the basis of the dietary n6/n3 ratio and DHA/EPA supplementation (Figure 1). The n6/n3 ratio was ~7 for the CON and HF groups, ~50 for the N6-HF group, and ~1 for the DHA-HF group (Table 1 and Table 2). The experiments lasted for 22 weeks, with 2 weeks of acclimation and 20 weeks of treatment. The rats were kept in polycarbonate cages and housed under the conditions of a 12 h light/dark cycle at 25 ± 2 °C. Access to food and water (tap water) was unlimited to all. Body weight was measured daily at the same time (8:00 a.m.) using a digital scale.

### 2.2. Sample Collection

After 20 weeks, rats were sacrificed by exsanguination in deep anaesthesia (Narketan, 80 mg kg^−1^ b.m. and Xylapan,12 mg kg^−1^ b.m., i.p., Vetoquinol, Bern, Switzerland). Blood samples were collected by cardiac puncture into serum tubes, and serum was obtained after centrifugation at 1500× *g* for 5 min. The abdominal adipose tissue was excised promptly after the animals were sacrificed, divided, and stored in an RNA-preserving agent (RNAlater, Thermo Fisher Scientific, Waltham, MA, USA). 

### 2.3. Serum Biochemistry

The fasting blood glucose levels were analysed by glucose meter Accu-Chek Go [13]. The levels of triglycerides and total cholesterol were analysed by an automatic analyser (SABA-18, Analyser Medical System, Roma, Italy). Commercial rat insulin ELISA kit was used to determine insulin concentration (Rat insulin kit ELISA, Mercodia, Upsalla, Sweden). Insulin sensitivity was measured as HOMA-IR [14] and QUICKI [15] indices, using the following equations:

(1) HOMA IR=(fasting insulin×fasting glucose)/22.5

(2) QUICKI=1/(log(fasting insulin)+log(fasting blood glucose))

### 2.4. Determination of Oxidative Status Measurement

Following a previously described method, the HPLC method was used for analysing malondialdehyde (MDA) content as MDA-TBARS (thiobarbituric acid-reacting substances) [16].

Briefly, an aliquot of 20 μL of serum was injected onto a Shimadzu LC-2010CHT with an Inert Sustain C18 column (4.6 mm 150 mm, 5 μm particle size; GL Sciences, Tokyo, Japan). The standard curve was prepared using 1,1,3,3-tetraethoxypropane. MDA-TBARS were expressed as μM.

### 2.5. Lipid Extraction and Quantitation of Fatty Acid Composition

Total lipids from the tissue were extracted using a chloroform/methanol mixture (2:1, *v*/*v*) [17]. After extraction, the lipids were dried under N_2_, dissolved in the same mixture (150 μL), with the addition of 0.3 mg/mL butylated hydroxytoluene (BHT), and stored at −80 °C. The analysis of fatty acid was performed by GC–MS (QP2010 Ultra, Shimadzu, Kyoto, Japan), with BPX70 capillary column (0.25 mm internal diameter, 0.25 μm film thickness, 30 m long, SGE, Austin, TX, USA). Analytical conditions were described previously [18]. The internal standard used for quantification of fatty acid methyl esters was nonadecanoic fatty acid (C19:0). The results of fatty acid composition were expressed as the percentage of total fatty acids.

### 2.6. Western Blotting Analysis

Total proteins were extracted by homogenisation in lysis buffer (RIPA lysis buffer, EMD Millipore, Billerica, MA, USA), containing a protease inhibitor cocktail (SIGMAFAST Protease Inhibitor Tablets, Sigma Aldrich, Taufkirchen, Germany). The samples were centrifuged for 30 min at 14,000× *g* at 4 °C, and the supernatant was collected into clear tubes. BCA assay (Sigma Aldrich, Taufkirchen, Germany) was performed for the determination of the protein concentration. Denaturation of 20 μg of total proteins in Laemmli SDS loading buffer was performed at 95 °C for 5 min. After loading, 10% SDS polyacrylamide gel electrophoresis was carried out and then proteins were transferred to nitrocellulose membranes. Afterwards, the membranes were blocked in blocking buffer (ImmobilonBlock—CH (Chemiluminescent Blocker), MerckKGaA, Darmstadt, Germany) and incubated overnight at 4 °C with primary antibody dissolved in ImmobilonBlock—CH (Chemiluminescent Blocker). After the membranes were washed three times for five minutes each with Tris-buffered saline with 0.5% Tween 20 (TBST), they were incubated in the corresponding secondary antibody for 1 h at room temperature. After being washed again three times with TBST, the membranes were processed for enhanced chemiluminescence (ECL) Clarity Max Western ECL Substrate (Bio-Rad Laboratories, Hercules, CA, USA) and captured using Odyssey Fc (LICOR, Bad Homburg, Germany). The primary antibody used was anti-adiponectin (dilution 1:500, Anti-Adiponectin antibody: ab133347, Abcam) and corresponding secondary HRP-conjugated IgG antibody (dilution 1:1000, mouse anti-rabbit IgG-HRP: sc-2357, Santa Cruz Biotechnology). Equality of loading and normalisation of the bands was assessed by using Amidoblack. 

### 2.7. RT-qPCR Analysis

Total RNA was extracted from frozen adipose tissue using SV Total RNA Isolation System (Promega GMBH, Mannheim, Germany), according to the manufacturer’s instructions. The quantity and purity of the isolated RNA samples were checked by spectrophotometry (BioDrop μLITE, BioDrop, Cambridge, UK). Total RNA was subjected to a one-step RT-qPCR reaction using One-Step SYBR PrimeScript RT-PCR Kit II, according to the manufacturer’s manual (Perfect Real Time, TaKaRa Bio Inc., Shiga, Japan). The qPCR was performed using a Stratagene MxPro3005 (Agilent Technologies, Santa Clara, CA, USA and Mississauga, ON, Canada) thermocycler. Melt curve analyses verified the formation of a single desired PCR product in each PCR reaction. The expression level of each sample was normalised against the expression of β-actin as the internal control. The ratio of the relative expression of target genes to the housekeeping genes was calculated using the comparative *C*_T_ method 2^(−ΔΔCT)^, and values were normalised to β-actin and cyclophilin levels [19]. All reactions were performed in triplicate for each sample. The primer sequences are listed in Table 3.

### 2.8. Statistical Analyses

The GraphPad Prism 8 program was used for statistical analysis of the obtained experimental results. Shapiro–Wilk test was used to test data normality. Data are expressed as means ± SD. A one-way ANOVA, followed by post hoc Tukey tests, was used to compare the treated groups to the control. The standard deviations of the 2^−ΔΔCT^ equation for the tested gene expression were determined according to the established procedure [19]. Significant differences set up was at *p* < 0.05 and *p* < 0.01.

## 3. Results

### 3.1. Body Weight and Insulin Resistance Confirmation

**Figure 2:**
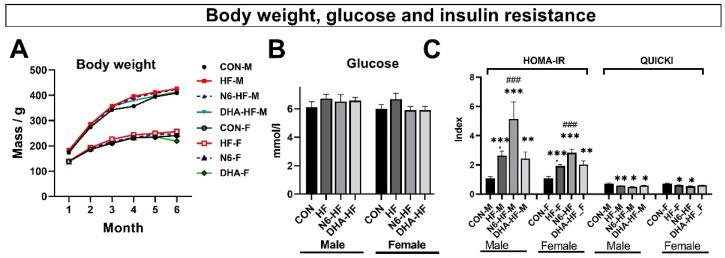
The influence of the treatments on body weight (**A**) and fasting blood glucose values (**B**). Insulin sensitivity was assessed as insulin concentration and insulin sensitivity indices, HOMA-IR, and QUICKI (**C**). Values are means ± SD. * *p* < 0.05, ** *p* < 0.01, *** *p* < 0.001 for HF, N6-HF, and DHA-HF groups versus the CON group and ### *p* < 0.001 for N6-HF, and DHA-HF groups versus the HF group. All values were compared between the groups within each sex.

After 20 weeks, the animals assigned to different experimental diets did not show significant differences in body weight or blood glucose level (Figure 2A,B). Although there were no changes in the blood glucose level, insulin sensitivity expressed as HOMA-IR and QUICKI indices were affected in all fructose supplemented groups (Figure 2C). Significant differences in insulin sensitivity (*p* < 0.001) were observed between the control group (CON) and all the other experimental groups. Additionally, fructose treatment in the male N6-HF group worsened insulin sensitivity indices, compared with the group with basal n6/n3 ratio (Figure 2C).

### 3.2. Biochemical Parameters

**Figure 3:**
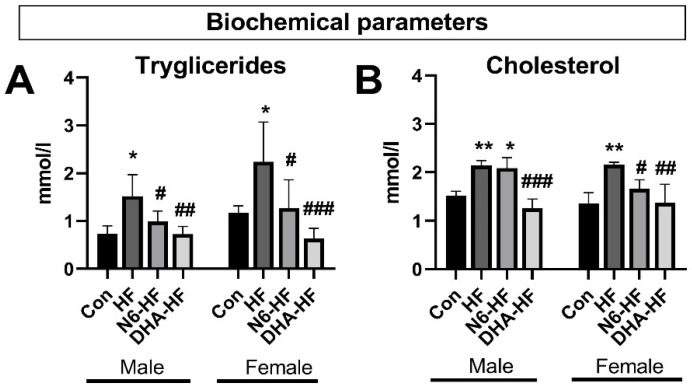
Influence of treatments on serum triglycerides (**A**) and cholesterol level (**B**). Values are means ± SD. * *p* < 0.05, ** *p* < 0.01, for HF, N6-HF, and DHA-HF groups versus the CON group and # *p* < 0.05, ## *p* < 0.01, ### *p* < 0.001 for N6-HF and DHA-HF groups versus the HF group.

The serum triglycerides in females of the HF group showed an increase, in comparison with male rats fed an HF diet (Figure 3). When the fructose diet also contained DHA/EPA supplementation, the high levels of serum triglycerides and cholesterol concentrations returned to levels similar to those of the control group (*p* < 0.01).

### 3.3. Lipid Peroxidation and NRF 2 Expression 

**Figure 4:**
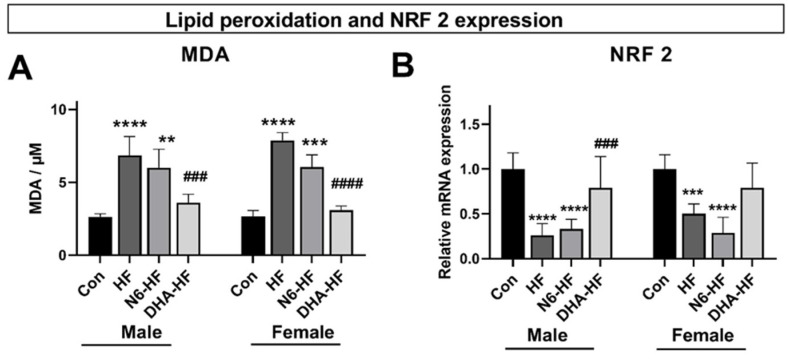
Effects of treatments on the lipid peroxidation measured as MDA-TBARS value in serum (**A**) and antioxidative response as mRNA expression of NRF 2 in adipose tissue (**B**). Values are means ± SD., ** *p* < 0.01, *** *p* < 0.001, **** *p* < 0.0001 for HF, N6-HF, and DHA-HF versus the CON group and ### *p* < 0.001, #### *p* < 0.0001 for N6-HF, and DHA-HF versus the HF group.

After 20 weeks of administrating a high-fructose diet, the values of MDA-TBARS were increased in the serum of the HF and N6-HF groups but not in the DHA-HF group. The results were the same for both sexes (Figure 4A). A significant decrease (*p* < 0.001) in MDA-TBARS serum values was observed in the DHA-HF group, compared with the N6-HF and HF groups in both sexes. The mRNA expression of NRF 2 decreased significantly in the HF and N6-HF groups in comparison with the CON group, in both males and females (*p* < 0.001) in adipose tissue (Figure 4B). In contrast, the DHA-HF group had NRF 2 expression similar to the values of the CON group.

### 3.4. Expression of Inflammatory Markers and Adiponectin

**Figure 5:**
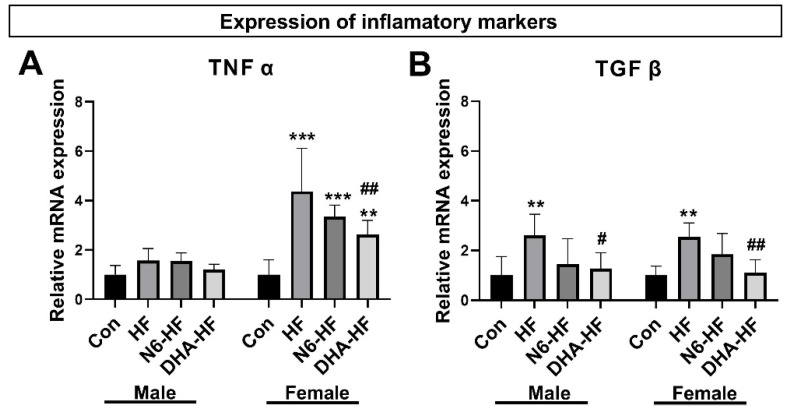
The gene expression of the inflammation markers TNF α (**A**) and TGF β (**B**) in adipose tissue. Values are means ± SD, ** *p* < 0.01, *** *p* < 0.001, for HF, N6-HF, and DHA-HF versus the CON group and # *p* < 0.05, ## *p* < 0.01, for N6-HF and DHA-HF versus the HF group.

In the adipose tissue, we investigated the expression of inflammatory adipokines, tumour necrosis factor α (TNF α), and transforming growth factor β (TGF β) (Figure 5A,B). An increase in TNF α expression was observed in the high-fructose-fed groups, compared with the CON group in female rats (*p* < 0.01), while in male rats, the difference did not reach a significant level. The DHA-HF female group had significantly lower TNF α expression in comparison with the HF female group (*p* < 0.01).

The expression of TGF β was significantly increased (*p* < 0.01) in the HF group, in both female and male rats. In the DHA-HF group, TGF β gene expression was significantly lower in comparison with the HF group, in males and females (*p* < 0.05, *p* < 0.01, respectively).

**Figure 6:**
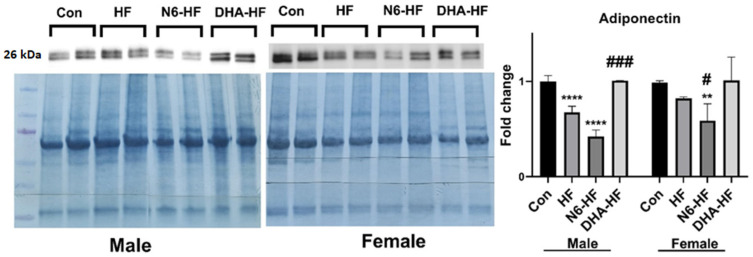
Adiponectin protein abundance in adipose tissue was determined by Western blotting relative to total protein. Values are means ± SD., ** *p* < 0.01, **** *p* < 0.0001 for HF, N6-HF, and DHA-HF groups versus the CON group and # *p* < 0.05, ### *p* < 0.001 for N6-HF and DHA-HF groups versus the HF group.

The influence of high-fructose treatment was clearly visible on the expression of adiponectin protein abundance in adipose tissue (Figure 6). Adiponectin expression significantly decreased in the HF and N6-HF groups in male and female rats but not in the DHA-HF group (*p* < 0.001). The DHA-HF group also had higher adiponectin expression compared with the N6-HF and HF groups.

### 3.5. Delta 9 Desaturase Expression (Δ9D)

**Figure 7:**
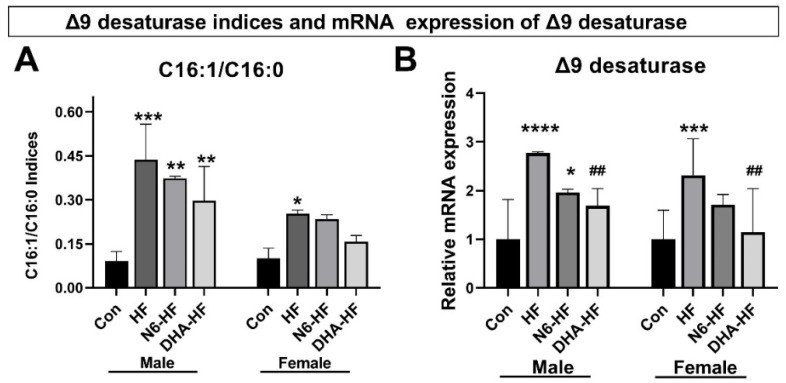
Effects of treatments on the desaturase indices C16:1/C16:0 (**A**) and mRNA expression of the Δ9 desaturase (**B**) in adipose tissue. Values are means ± SD. * *p* < 0.05, ** *p* < 0.01, *** *p* < 0.001, **** *p* < 0.0001 for HF, N6-HF, and DHA-HF groups versus the CON group and, ## *p* < 0.01, for N6-HF and DHA-HF groups versus the HF group.

Quantitative PCR revealed a significant increase in Δ9 desaturase (stearoyl-CoA desaturase-1) gene expression in adipose tissue in the high-fructose-fed male and female rats (Figure 7B). The DHA supplementation in the diet showed a lowering effect on Δ9 desaturase expression in adipose tissue. The lowering effect was more pronounced in female animals. The desaturation index (C16:1/C16:0) showed an increase in DNL in the HF group and N6-HF group (Figure 7A). The positive effect of DHA supplementation was observed in the decrease in Δ9 desaturase activity.

### 3.6. Adipose Tissue Fatty Acid Profile

**Figure 8:**
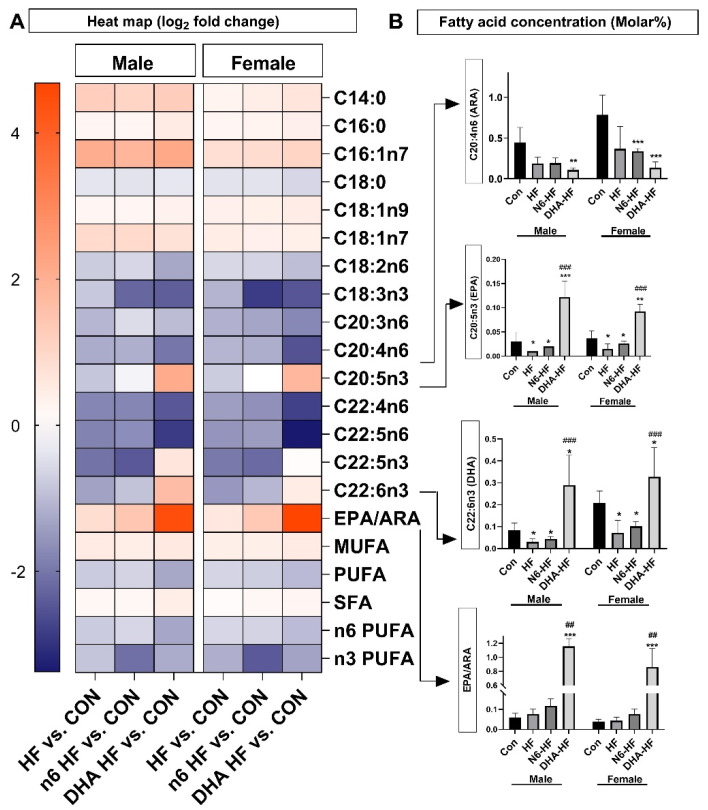
The influence of the treatments on the fatty acid profile of the adipose tissue is presented as a heat map. Values are means ± SD. * *p* < 0.05, ** *p* < 0.01, *** *p* < 0.001, for HF, N6-HF, and DHA-HF groups versus the CON group and, ## *p* < 0.01, ### *p* < 0.001 for N6-HF and DHA-HF groups versus the HF group.

The fatty acid composition of the adipose tissue is presented in a heat map (Figure 8). The characteristic differences were an increase in the content of monounsaturated fatty acids (MUFA) C16:1n7, C18:1n9, and C18:1n7. The influence of the treatments was also observed in the content of important n6 and n3 PUFAs. The content of arachidonic acid (ARA, C20:4n6) was significantly decreased in all treated groups, in both males and females (*p* < 0.001). The content of EPA (C20:5n3) was significantly higher in the DHA-HF group (*p* < 0.001). In addition, the content of EPA decreased significantly in the HF and N6-HF groups (*p* < 0.05), compared with the CON group, in both sexes. Changes in ARA and DHA content in the DHA-HF group resulted in a very high EPA/ARA ratio in this group (Figure 8). The DHA-HF group showed a significantly (*p* < 0.01) higher EPA/ARA ratio in comparison with the CON group in both sexes. The content of DHA (C22:6n3) decreased significantly in the N6-HF and HF groups in comparison with the CON group, in both sexes (*p* < 0.05). Additionally, the DHA-HF group had a significantly higher content of DHA compared with the HF and N6-HF groups.

## 4. Discussion

Obesity is nowadays widely accepted as a significant health problem worldwide, and many studies are trying to elucidate the mechanisms associated with the diet and the onset of obesity. However, there are still many unknowns connected with this highly relevant and timely topic. The association of obesity and dysfunction of lipid metabolism is acknowledged as an important factor related to the genesis of metabolic syndrome. In the present study, long-term fructose administration via drinking water increased fasting insulin values without an increase in the fasting glucose, indicating the early stages of β-cell dysfunction. This finding is not atypical in the early stages of obesity and metabolic syndrome studies characterised by hyperinsulinemia without hyperglycaemia [20]. Different stages of progression to diabetes, characterised by different relationships between insulinemia and glycaemia, are described in human diabetes [21]. Factors that could contribute to the insulin sensitivity indices include study duration, dosage, and the type of diet used to induce obesity (fructose, sucrose, and fat), weight gain, age, and baseline insulin sensitivity [22]. In addition, insulin sensitivity, determined by measuring HOMA-IR and QUICKI indices, was affected in all fructose treated groups and in both sexes. Nevertheless, from the results of this study, it is evident that a high n6/n3 ratio has a worse effect on insulin sensitivity. It should be noted that insulin sensitivity expressed as HOMA-IR and QUICKI indices cannot be considered as a replacement for direct measurement of insulin resistance, but both provide a reliable approximation of direct measures of insulin resistance in rodents and humans [23]. 

Although unexpected, a high-fructose diet did not cause a significant increase in body weight, a finding similar to some previous studies [24,25,26]. Nevertheless, an important observation was that in human studies with fructose-supplemented diets, in which body weight did not increase, the deleterious effects on inflammation were still present [27,28].

Previous research established that fructose overconsumption causes the accumulation of visceral fat [22,29], increases the production of pro-inflammatory adipokines, and activates oxidative stress [30,31]. To evaluate oxidative stress as a key component of metabolic syndrome, the concentration of MDA, as a biomarker of lipid peroxidation, was measured [32,33], as well as NRF 2 expression, which is a key transcription factor with a central role in the endogenous antioxidant cellular defence system. An increase in the MDA concentration is a regular finding in various metabolic disorders [34], and therefore, a marked increase in the serum MDA values was expected in this trial (Figure 3A). Nevertheless, an interesting finding was that a low n6/n3 ratio attenuated an increase in the MDA content, showing that DHA can exhibit antioxidative properties. The antioxidative properties of DHA are still controversial due to the differences in the experimental designs and dose-dependent effects (antioxidative in the smaller dosage and prooxidative in the higher dosage) [35,36], and because the underlying molecular mechanisms are not completely elucidated [37].

As previously established, the changes in NRF 2 expression are associated with different metabolic disorders and inflammation [38]. In this study, fructose significantly decreased the NRF 2 expression. Decreased NRF 2 activity caused by fructose over-feeding was previously observed as a consequence of Keap1 upregulation, which results in inactivation of the NRF 2 antioxidant pathway [39]. The potential modulatory effect of low n6/n3 ratio on NRF 2 expression and overall antioxidative defence was clearly visible in the trial because groups with a low n6/n3 ratio reverted the NRF 2 expression to the values of the control animals [40].

The results showed an increase in the expression of measured inflammatory markers, TNF α, and TGF β. These results are in accordance with the well-known fact that TNF α and TGF β are increased in obesity and related metabolic diseases [41,42]. Moreover, in obesity, infiltration of macrophages into adipose tissue results in the increased secretion of proinflammatory cytokines which further contributes to insulin resistance [43]. It is important to note that a low n6/n3 fatty acid ratio decreased the expression of measured inflammatory markers to the value of the control animals. Although attenuation of the increased expression of inflammatory markers was not visible in all groups, it is evident that a low n6/n3 ratio can modulate chronic low-grade inflammation even in the early hyperinsulinemic stage of metabolic syndrome. In recent decades, it has become obvious that the long-chain n3 fatty acids (particularly DHA and EPA) could ameliorate inflammation associated with different disorders, but the underlying molecular mechanisms are still heavily investigated [35]. The key mechanism of DHA and EPA anti-inflammatory action is a decrease in the production of proinflammatory eicosanoids originating from arachidonic acid [44] and an increase in the production of anti-inflammatory molecules originating from EPA and DHA (e.g., resolvins, protectins, maresins) [45]. In addition, DHA and EPA also have anti-inflammatory effects that are not directly related to eicosanoid production including the influence on the cell surface expression of adhesion molecules [46], inhibition of the synthesis of inflammatory cytokines [47,48], and modulation of the inflammatory gene expression [49,50].

In addition to inflammatory markers, the expression of adiponectin also reverted to physiologic values in rats with a low n6/n3 ratio. Adiponectin is a specific hormone with insulin-sensitising and anti-inflammatory activity and plays a role in glucose and lipid homeostasis [51,52]. Thus, imbalance in adiponectin production in the adipose tissue and in relation to oxidative stress could contribute to the development of insulin resistance and consequently metabolic syndrome [22].

Diets enriched with easily absorbable carbohydrates, such as fructose, are able to potently stimulate de novo lipogenesis DNL [53]. We measured DNL indirectly by calculating an index based on the fatty acids that are synthesised de novo: palmitoleic (C16:1n7) and oleic (C18:1n9). The results showed increased DNL in all high-fructose-fed groups in comparison with the CON group. C18:1n9 and C16:1n7 and total MUFA were found in increased quantities in the adipose tissue of the high-fructose-fed groups, indicating an increase in the Δ9 desaturase activity. Furthermore, Δ9 desaturase catalyses the conversion of C16:0 and C18:0 fatty acids into their monounsaturated forms (C16:1n7 and C18:1n9) [54,55]. We further investigated the calculated indices with RT-qPCR, which confirmed an increase in the quantity of Δ9 desaturase mRNA. The low n6/n3 ratio decreased Δ9desaturase expression, which is in agreement with the well-established role of PUFAs on the expression of Δ9 desaturase in rodents [18,56,57].

In contrast to MUFA, PUFA content was decreased after fructose treatment in both sexes. The content of essential fatty acids, linoleic and linolenic acid, was also decreased in other investigations with a high content of carbohydrates [18,58]. Linoleic and linolenic acid are precursors for the synthesis of other important n3 and n6 fatty acids in the series of steps of desaturation, elongation, and β oxidation. Therefore, a decrease in either of these two acids results in the decreased content of important PUFAs—namely, ARA, EPA, and DHA [18,59].

Considering PUFA content, EPA and DHA content decreased in the fructose-treated groups, except for the group with the low n6/n3 ratio. This was expected, as the low n6/n3 ratio group had a high content of DHA and EPA in their diet.

The content of arachidonic acid (ARA) significantly decreased in all fructose-treated groups. Arachidonic acid is a precursor for prostaglandins, thromboxanes, and leukotrienes, and is considered a predominant proinflammatory PUFA [60]. In contrast, EPA is considered anti-inflammatory because it is a precursor for less inflammatory eicosanoids and different resolvins, and is a competitive inhibitor of ARA for oxygenases (cyclooxygenase and lipoxygenase). Consequently, the EPA/ARA ratio could be a significant indicator of the degree of inflammatory processes and the development of inflammatory diseases [61].

## 5. Conclusions

In the present research, we established that the dietary treatment with 15% fructose-induced significant changes in the expression of inflammatory markers, lipid peroxidation, and fatty acid profile (decrease in EPA/ARA ratio and increase in MUFA content) in rat abdominal adipose tissue. These changes undoubtedly point to the susceptibility to chronic low-grade inflammation as a hallmark of many important metabolic and other chronic diseases. The addition of high quantities of dietary DHA and EPA to the fructose-challenged rats, which resulted in a drastic decrease in the n6/n3 ratio, showed a protective effect in a defence mechanism against insulin resistance, inflammation, oxidation, dyslipidaemia, and changes in the fatty acid composition of the rat adipose tissue. In light of the fact that adipose tissue is a source of important PUFAs for other organs, as well as a source of inflammatory cytokines, these changes could have an important role in the development of many chronic diseases in humans and animals. Therefore, all dietary interventions, including supplementation with DHA and EPA and decreasing the n6/n3 ratio, which could attenuate these pathological changes in the adipose tissue, represent interesting strategies in preventing metabolic diseases for which an unmet medical need still exists.

## Figures and Tables

**Table 1:** Nutrient composition of the experimental diet (dry weight basis).

Nutrients (% Unless Otherwise Stated)	Exp. Diet
Crude protein	20.2
Crude fat	4.9
Crude fibre	4.9
Ash	6.7
Calcium	1.0
Phosphorus	0.8
Lysine	1.2
Methionine	0.4
ME (MJ)^2^	12.5

**Table 2:** Fatty acid composition (% of fatty acids) of diets supplemented to the rats.

Fatty Acids	CON & HF	N6	DHA
Palmitic (C16:0)	7.15	7.36	15.20
Stearic (C18:0)	2.76	2.21	4.30
Oleic (C18:1n9)	28.97	29.09	31.21
Linoleic (C18:2n6)	52.25	58.69	26.78
Linolenic (C18:3n3)	7.35	1.20	0.87
Eicosapentaenoic (C20:5n3)	nd	nd	9.15
Docosahexaenoic (C22:6n3)	nd	nd	10.98
n6/n3 ratio	7.11	48.91	1.28

nd, below quantification level.

**Table 3:** Primer sequences and cycling conditions for polymerase chain reactions to evaluate the impact of the treatment in gene expression.

Gene	Sequence 5′-3′	Access. No.	Ann. Tm.	Cycles
TGF β	F: AAT ACG TCA GAC ATT CGG GAA GCA	NM_021578.2	60 °C	40
	R: AAT ACG TCA GAC ATT CGG GAA GCA			
TNF α	F: CAC CAC GCT CTT CTG TCT ACT GAA C	NM_012675.3	60 °C	40
	R: CCG GAC TCC GTG ATG TCT AAG TAC T			
Δ9D	F: ACA TTC AAT CTC GGG AGA ACA	NM_139192.2	60 °C	40
	R: CCA TGC AGT CGA TGA AGA AC			
NRF 2	F: CAC ATC CAG ACA GAC ACC AGT	NM_031789.2	60 °C	40
	R: CTA CAA ATG GGA ATG TCT CTG C			
β-actin	F: CAT TGT CAC CAA CTG GGA CGA TA	XM_039089807.1	60 °C	40
	R: GGA TGG CTA CGT ACA TGG CTG			
Cyclophilin	F: GGA TGG CAA GCA TGT GGT CTT TG	M19533	60 °C	40
	R: CTT CTT GCT GGT CTT GCC ATT CCT			

## Data Availability

Data is contained within the article.
